# Advanced theoretical-applied model based on the PD approach in the light of healthcare-associated infections: what have we achieved so far?

**DOI:** 10.3389/fpubh.2024.1291551

**Published:** 2024-02-12

**Authors:** Ricky Cohen, Anat Gesser-Edelsburg

**Affiliations:** ^1^Cheryl Spencer Department of Nursing and the Health and Risk Communication Research Lab, University of Haifa, Haifa, Israel; ^2^Head of the Health Promotion Program and Head of the Health and Risk Communication Lab, School of Public Health, University of Haifa, Haifa, Israel

**Keywords:** positive deviance approach, healthcare-associated infections, infection prevention and control, health professionals, theoretical-applied model

## Abstract

Healthcare-associated infections remain a persistent concern despite decades of research and intervention efforts. Adherence to infection prevention and control guidelines by health professionals remains a challenge, necessitating innovative strategies. The Positive Deviance (PD) approach, rooted in harnessing localized solutions, holds promise but lacks comprehensive frameworks and empirical validation to bolster its theoretical underpinnings. This perspective article serves a dual purpose: first, to examine the unique challenges of applying the PD approach in the context of HAIs; and second, to introduce a robust theoretical-applied model developed in response to these challenges. This article addresses these gaps through a multi-faceted model developed in a mixed-methods study across three Israeli governmental hospitals and comprises four essential components that address the identified gaps in existing research. This article enriches the dialog on PD’s applicability in HAIs by providing a robust model that not only offers solutions but reshapes the approach to healthcare hygiene and safety. It responds to critical gaps highlighted in the literature, offering tailored interventions by practical, context-specific solutions to improve adherence to IPC guidelines in the long term. Methodological clarity is also a key focus, offering a toolkit for practical implementation. This bottom-up approach empowers HPs to drive change, fostering a culture of innovation and improvement in healthcare settings.

## Introduction

For over three decades, healthcare-associated infections (HAIs) have remained a pressing concern, with adherence to infection prevention and control (IPC) guidelines by health professionals (HPs) being an area of particular focus. Despite extensive research and numerous intervention programs, efforts to improve long-term adherence have yielded limited results (1–3). Concurrently, an emerging body of evidence highlights the potential of integrating theory-based behavioral components into these interventions to enhance their sustainability (4, 5).

Among the diverse range of socio-behavioral strategies under investigation, the Positive Deviance (PD) approach stands out for its unique focus on harnessing community- or organization-based solutions. Rooted in the principle of identifying and learning from individuals or groups who have successfully navigated challenges under similar constraints, the PD approach aims to unearth localized, effective practices for broader implementation (3, 6–8).

However, there exist gaps in the current literature on PD. Herington and Fliert’s seminal 2018 review highlighted three critical needs: the development of context-specific frameworks, a foundational set of guiding principles for the PD approach, and the execution of empirical research to bolster its theoretical underpinnings (9).

Addressing these explicit gaps, our research engaged in a comprehensive, mixed-methods study conducted from January 2017 to November 2020 across three governmental hospitals in Israel, involving a diverse range of 250 healthcare professionals. This study not only applied the PD approach to mitigate the prevalence of HAIs but also aimed to contribute to its theoretical and methodological scaffolding (10–14).

The present article serves a dual purpose: first, to examine the unique challenges of applying the PD approach in the context of HAIs, as underscored by Herington and Fliert’s review; and second, to introduce a robust theoretical-applied model developed in response to these challenges. This model aligns closely with Herington and Fliert’s recommendations and comprises four essential components that address the identified gaps in existing research. Through this comprehensive examination, we aim to contribute not only to the theoretical understanding of the PD approach but also to offer practical, context-specific solutions to improve adherence to IPC guidelines in the healthcare setting.

In light of this, our research provides a targeted response to the key questions and gaps identified by Herington and Fliert, offering a pathway for more effective and tailored interventions in the fight against HAIs. This article aims to enrich both the theoretical and practical dialog on the applicability and expansion of the PD approach in the realm of HAIs.

## Challenge 1: how can HPs maintain a hygienic behavior routine in any situation over time?

*The problem* is that existing IPC guidelines do not address the “gray area” barriers. We coined the phrase “gray area” for the first time to define the situations where staff members are unaware of what is required of them, which then leads to confusion, frustration, and uncertainty. Gray areas encompass the variety of situations on the care continuum that are not addressed in the accepted guidelines, and where staff members are unsure of how to proceed (14). It gives a social proof by recognizing the behavioral difficulties that staff members have reported. Those difficulties prevent them from adhering to IPC guidelines or retrospectively interpreting different situations on the care continuum that lead to different behaviors. Gould et al. (1) in their new paper titled “The problem with ‘My Five Moments for Hand Hygiene’,” claim that it is not always possible to implement the “Five Moments” for all patients all the time. Patients have widely differing needs in diverse settings, and the “Five Moments” do not adapt well to all these many differences and may overlook barriers that can reduce hand hygiene adherence. These findings are also consistent with a rapid qualitative evidence synthesis, recently published by Houghton et al. (15) in the light of COVID19, which found that HPs often feel unsure as to how to adhere to local guidelines when they were lengthy and ambiguous, or do not reflect national or international guidelines. They could feel overwhelmed because local guidelines are constantly changing, which leads to increased workloads and fatigue. Since contemporary literature began to internalize that the range of dynamic situations that occur on the care continuum in different settings with different patients, it is clear that one guideline does not fit all. It intertwines with the other well-known barriers (individual, environmental, etc.) and helps interpret the behavioral difficulties of the staff members, which clarifies the rationale for complexity.

*The solution* is to use the PD approach to identify PD practices derived from community members and implemented in a medical unit’s work environment. PD practices are like “unwritten guidelines” since they complement the missing parts of formal guidelines. In our previous article we described the deconstruction process – how we disassemble central line insertion guidelines, and then creatively reassemble them by using PD solutions. In fact, in this example, several physicians from the intensive care unit offered 23 unique solutions throughout the process of inserting a central catheter, which did not originally appear in the official guidelines. This new interpretation by the PDs provides an expansion, which connects and anchors the current guidelines to the ground and thus complements the missing parts in the formal guidelines, therefore they are used as “unwritten guidelines” (10).

## Challenge 2: how to create HPs engagement when responsibility is transferred from the top to the community itself?

*The problem* is that most intervention programs in the field of IPC do not last long and are not community based (1–3).

*The solution* is identifying the PD individuals, building social network maps and performing collaborative simulations by HPs. Unsurprisingly it seemed that as we progressed throughout the study and identified more and more PD practices, the PD individuals were able to address gray area barriers that most HPs had raised in the first phase of the study. The PD individuals were naturally the ones who felt these barriers took them out of the comfort zone and led them to think outside the box and act differently than their colleagues. In order to identify the PDs, we used face to face interviews with staff members, while using snowball sample. Staff members were asked to identify other staff, who in their view is a PD (according to the definitions given to them). During that process, we built the social network map that helped us understand the “roles” of the team members within the departments, and more importantly understand the staff members’ points of view, how they perceived the roles of their members within the network. Study findings indicate that from the first time an individual is asked to refer to another, it causes them to become engaged, connected, and visible. Moreover, asking them to share their wisdom with others, empowers them and causes them to “acting out.” They feel their practice is important, has meaning, and contributes to infections prevention; then they enlist in the process. Building social network maps within health systems, and identifying the most prominent people through their multitude of connections on the social map, accelerates HPs engagement and improves organizational performance over the long run (16, 17).

Schaufeli and colleagues (18) defined engagement ‘as a positive, fulfilling, work-related state of mind that is comprised by vigor, dedication, and absorption. Vigor is a high level of energy while working, willingness to invest effort in one’s work, and persistence even in the face of difficulties. Dedication is characterized by a strong psychologic involvement in one’s work and by a sense of significance, inspiration, pride, and challenge. Absorption refers to total concentration on work. In the last decade, there have been rapidly-increasing, evidence-based advancements in the field of work engagement, which has become an important consideration for many organizations, since it leads to improvement in individual and organizational outcomes, such as health and well-being, performance, and safety (19–22).

The study by Ancarani et al. (23) emphasizes the importance of using approaches that take into account employee engagement. The researchers investigated links between organizational climates and work engagement in a sample of public hospitals, which showed positive associations between work engagement and a climate promoting worker’s autonomy, empowerment, and well-being. Moreover, work engagement is associated with feelings of significance, keenness, passion, motivation, and gratification, and indicates an enduring emotional inspirational state, rather than a momentary and specific emotional condition. Furthermore, from a systematic review by Knight et al. (21) who sought to examine which interventions-based engagement were found to be most effective, it emerged that 70% of the successful interventions were bottom-up. Bottom-up interventions involved encouraging individuals to proactively make changes themselves. They are most successful because they are driven by individual employees, who themselves initiate and make the changes. The findings of this review reinforce our claim that the engagement component is significant in predicting positive outcomes, and determine the success of intervention programs, especially those that are bottom up like PD.

Our research findings show it is possible to identify staff members who found creative and practical solutions, big and small, that are not written or recommended in the formal IPC guidelines but address “problematic” and vague situations on the care continuum they themselves raised. Because these solutions come from the community, it is very likely that people within the system will be more open to adapting them (24). Furthermore, disseminating new ideas from staff members creates an environment of eagerness to find even more constructive ideas, similar to the feelings Alessandro described in his study (23). The dissemination of PD practices was performed by PD individuals through learning simulations recorded on smartphone video (10, 13). We described step-by-step, how the simulations continued engaging staff members, and gives a reasoned explanation for the significance of the additional and variant PD practices. The simulations were accompanied by an open dialog in which participants were invited to ask questions and raise other ideas, thus adding value to both the demonstrators and the audience. Documenting the procedures by videotaping with smartphones were important for designing and developing activities to spread the PD solutions and help community members learn and practice the positive behaviors identified. Moreover, a readily available device raises the likelihood that other HPs will watch the filmed procedures (10, 13). Indeed, the interviewees reported they incorporated the demonstrated practices into their routine work, and 69.4% of them reported they changed their behavior in line with the PD intervention (11).

## Challenge 3: how to turn PD practices into positive norms?

*The problem* is that not everyone is a PD. It may be argued that if most community members are not identified as PD, how will it be possible to bring about behavioral changes in other HPs?

*The solution* is identifying “PD Booster”s – a new group that fully implemented the practices, and also added their own practices.

In the Post Intervention phase of the study, we aimed to examine which variables made PDs different from their peers? (Socio cognitive characteristics profile). To date, studies in the field have focused only on discovering PD practices and not on the PD individuals themselves. Findings indicated there were differences in perceived threat, external locus of control, and social learning. The common denominator of these three components are an enormous sense of accountability and responsibility to prevent infections among the PDs. We argue that this sense of accountability is the key element that distinguishes those who exhibit PD behaviors from those who do not. This claim underpins the deep understanding that everyone plays a role in preventing the transmission of infections. Although findings indicated that almost 70% of HPs reported full implementation of the PD, almost 17% of them reported adding their own practices. Thus, a circular diffusion was generated, that gained momentum within the network and encouraged some of them to add tips, which created a new group we coined the “PD boosters” (11).

## Challenge 4: how to translate the PD approach into an applied methodological tool?

*The problem* is that most of the literature that deals with the PD approach presents it as a concept or framework, and there is no detailed practical list of steps for professionals who want to adopt the approach.

*The solution* is presentation of methodological tools that can be applied step by step from the individual level to the organizational level.

To the best of our knowledge, so far, no articles have been published describing how and why PD interventions work (25), rather the literature has focused on the effectiveness of the approach by assessing reductions in HAIs (7, 17, 26–28). Therefore, we saw great importance in the methodological applied expansion we conducted in our study, which can allow for more detailed insights into the study quality and degree of intervention implementation. A detailed description of the intervention methodology, with its applied steps and tools, may greatly assist researchers and professionals implementing similar studies in a variety of healthcare settings (12). This point was also raised in the article by Zingg et al. (29) which presents the conclusions of 42 experts from the IPC field [Geneva infection prevention and control (IPC) – think tank 2017) who came together to develop an IPC vision, a plan on how to prevent antimicrobial resistance (AMR), and to agree on a road map for research and public health activities. Their findings suggest that most IPC studies have examined the effectiveness of intervention programs but not focused on practical programs for implementing the guidelines. Therefore, they recommend investing in methods to improve the implementation of evidence-based measures in different healthcare contexts (29), or development of detailed guidelines for designing interventions as suggested by Colquhoun et al. (19). To handle this challenge, we translated the positive deviance approach to an applied methodological tool, proposing step-by-step actions to mitigate barriers and minimize the existing ambiguity between the written guidelines to the work in the field, among HPs. The methodological tool is used as tool kit to find applicable solutions for each barrier identified through interviews, focus groups, and observations, to promote the use of PD practices that are not found in the official IPC guidelines (12).

## Discussion

**Figure 1:**
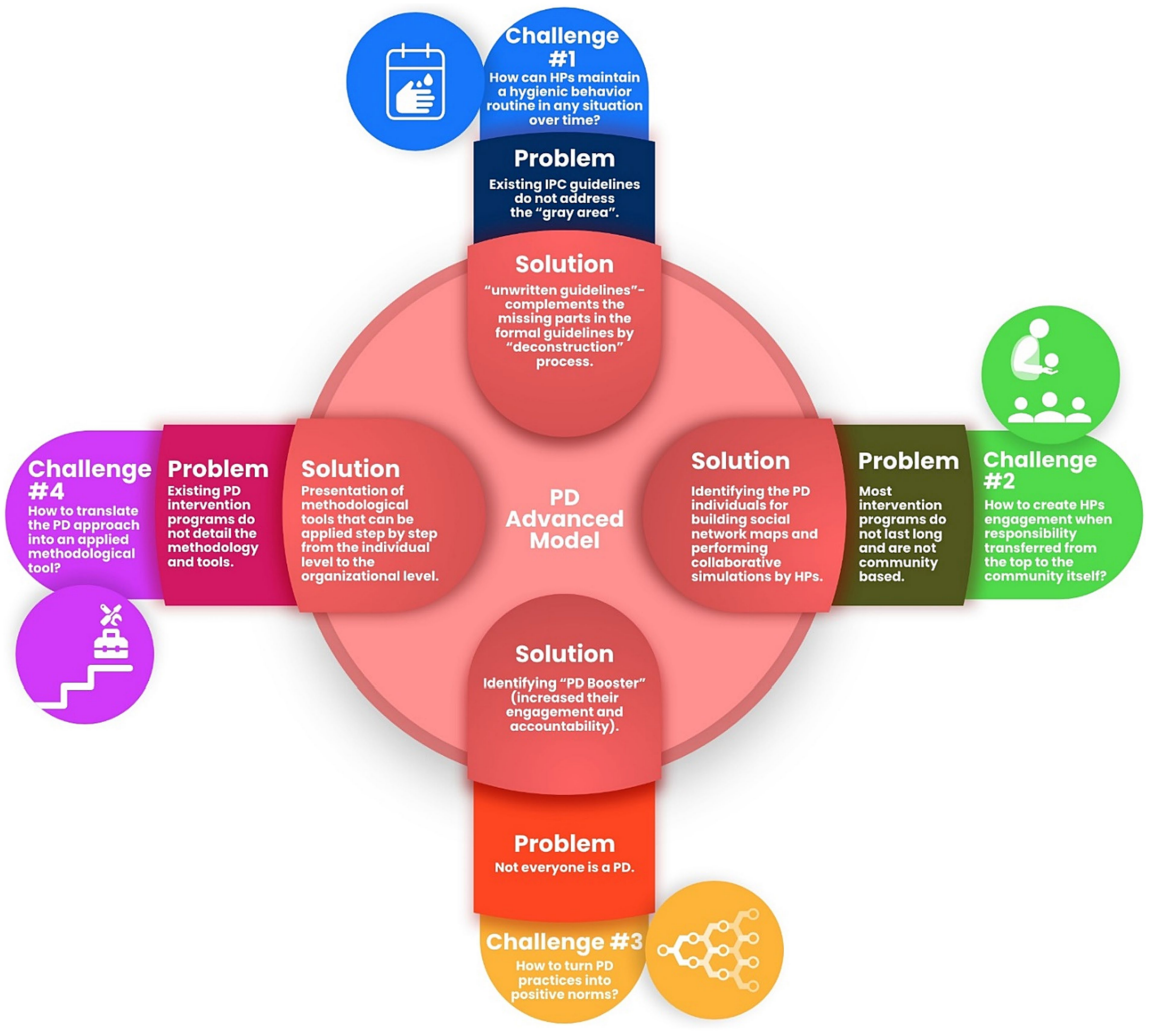
Advanced theoretical-applied model based on the PD approach.

Our research presents a multi-faceted model (Figure 1) that serves as a comprehensive framework IPC. Going beyond merely patching the gaps in existing IPC guidelines, this model provides a living, evolving blueprint tailored to the nuanced needs of healthcare professionals. It systematically addresses critical challenges, from how HPs can maintain hygiene in variable and often ambiguous situations, to how they can be authentically engaged in a community-based approach to IPC. The model introduces “gray areas” in IPC as areas of practice not covered by existing guidelines and proposes Positive Deviance (PD) as an innovative way to identify and implement effective, context-specific practices for such areas.

Furthermore, our research emphasizes the importance of HP engagement, highlighting how a community-driven approach can sustain long-term improvements. It introduces the concept of PD Boosters, staff members who not only implement best practices but also contribute their own innovative solutions, thereby nurturing an ongoing culture of excellence and accountability. This community-based, bottom-up approach is supported by an array of evidence, demonstrating its effectiveness in fostering a climate that encourages autonomy, empowerment, and well-being among HPs.

Finally, our model places a strong emphasis on methodological clarity, offering HPs a practical toolkit for translating PD insights into actionable steps. This is a critical contribution to the existing literature, which has largely focused on the effectiveness of PD but has been silent on how these interventions can be methodically implemented. Our model thus complements existing IPC guidelines and offers a flexible, adaptive approach that empowers HPs to be agents of change in their communities. This not only facilitates greater adherence to IPC measures but also fosters a self-renewing culture of innovation and improvement within healthcare settings.

By addressing these challenges through a community-based, bottom-up, and highly practical approach, our model advances the IPC field in a significant way. It offers not just solutions but a way of thinking and acting that could redefine the standards of healthcare hygiene and safety.

## Data availability statement

The raw data supporting the conclusions of this article will be made available by the authors, without undue reservation.

## Ethics statement

The studies involving humans were approved by the ethics committee at the Faculty of Social Welfare and Health Sciences, University of Haifa, confirmation number 392/17. The studies were conducted in accordance with the local legislation and institutional requirements. The participants provided their written informed consent to participate in this study. Written informed consent was obtained from the individual(s) for the publication of any potentially identifiable images or data included in this article.

## Author contributions

RC: Conceptualization, Data curation, Formal analysis, Investigation, Methodology, Visualization, Writing – original draft, Writing – review & editing. AG-E: Conceptualization, Data curation, Formal analysis, Funding acquisition, Investigation, Methodology, Writing – original draft, Writing – original draft.
